# Periodontal status and the efficacy of the first‐line treatment of major depressive disorder

**DOI:** 10.1002/cre2.492

**Published:** 2021-11-03

**Authors:** Silvana Jelavić, Žarko Bajić, Ivona Šimunović Filipčić, Ivana Jurčić Čulina, Igor Filipčić, Andrej Aurer

**Affiliations:** ^1^ Department for Extended Treatment and Palliative Care of Men University Psychiatric Hospital “Sveti Ivan” Zagreb Croatia; ^2^ Research Unit "Dr. Mirko Grmek" University Psychiatric Hospital "Sveti Ivan" Zagreb Croatia; ^3^ Department of Psychological Medicine University Hospital Center Zagreb Zagreb Croatia; ^4^ Solertia Dental Center Zagreb Croatia; ^5^ Department of Integrative Psychiatry University Psychiatric Hospital "Sveti Ivan" Zagreb Croatia; ^6^ Faculty of Dental Medicine and Health Josip Juraj Strossmayer University of Osijek Osijek Croatia; ^7^ Department of Psychiatry and Psychological Medicine School of Medicine, University of Zagreb Zagreb Croatia; ^8^ Department of Periodontology School of Dental Medicine, University of Zagreb Zagreb Croatia

**Keywords:** major depressive disorder, oral health, periodontal attachment loss, periodontal disease, selective serotonin uptake inhibitors, SSRI

## Abstract

**Objectives:**

The efficacy of treatment of major depressive disorder (MDD) is not satisfactory. Systemic inflammation may play an important role in MDD pathogenesis and treatment outcomes. Periodontal disease is the systemic inflammatory condition. Its prevalence may be as high as 45%. We aimed to assess the association of periodontal status with the outcome of 3‐month first‐line treatment of MDD with selective serotonin reuptake inhibitors.

**Material and Methods:**

We performed the prospective cohort study during 2018/2019 at Psychiatric Hospital “Sveti Ivan,” Croatia, on a consecutive sample of 43 patients. The outcome was the MDD symptoms severity measured using the Hamilton Depression Rating Scale‐17. The periodontal status was indicated by the clinical attachment loss (CAL).

**Results:**

Baseline periodontal status had a nonlinear significant and clinically relevant association with the MDD treatment outcome (*R* ^*2*^ change of the quadratic term = 0.12; *p* = 0.027). In patients with good baseline periodontal status the severity of MDD symptoms was significantly improved. When the value of CAL was ≥4.44 mm, indicating the worse periodontal status, further increase in baseline CAL was associated with the worsening of MDD treatment outcomes independently of the baseline depression severity and 14 sociodemographic and clinical predictors of treatment outcome.

**Conclusions:**

Periodontal healthcare is accessible, and should be utilize in an integrative, multidisciplinary approach not only for the sake of psychiatric patients' quality of life and prevention of periodontal disease, but for the sake of the outcomes of psychiatric treatment as well.

## 1 INTRODUCTION

Selective serotonin reuptake inhibitors (SSRI) are the recommended first line treatment for major depressive disorder (MDD) (National Institute for Health and Care Excellence (NICE), 2018), but their efficacy has been questioned (Jakobsen et al., 2017). Moreover, as much as two thirds of patients respond unsatisfactorily to the first treatment and one third not even to multiple interventions (De Carlo et al., 2016) while MDD remains among the most often causes of disability worldwide and the major risk factor for suicide (Vos et al., 2020). Various risk factors for poorer response has been identified, and among them the chronic physical illnesses and multimorbidities (Kraus et al., 2019). The risk for developing physical comorbidities is markedly higher in patients diagnosed with MDD, and particularly for comorbidities with a strong chronic inflammatory component (Firth et al., 2019). In return the inflammation has an important role in the pathogenesis of MDD (Majd et al., 2020), it is associated with the MDD severity (Firth et al., 2019), and treatment outcomes (Kraus et al., 2019; Liu et al., 2020). Periodontal disease is the exemplary inflammatory condition (Kinane et al., 2017) which may lead not only to local symptoms like the non‐reversible destruction of connective tissues of the periodontium and alveolar bone but also to cardiovascular, liver or Alzheimer's disease, chronic obstructive pulmonary disease, several types of cancer, diabetes, or schizophrenia (Kalakonda et al., 2016; Kitamura et al., 2019; Seitz et al., 2019). Its association with MDD has been extensively investigated, but the association remains controversial (Araújo et al., 2016; Kisely et al., 2016; Nascimento et al., 2019). However the prevalence of periodontal disease may be >40% of the ≥30 years‐old population (Eke et al., 2020) and its potential to affect the MDD treatment outcomes should not be overlooked. The most frequently used measure of periodontal tissue health is clinical attachment loss (CAL) (Savage et al., 2009). It is the loss of the periodontal support around a tooth. CAL <3 indicates no periodontitis or its mild form, CAL of 3 to 4 mm indicates stage II or moderate periodontitis, and CAL ≥5 mm indicates stage III or IV or severe periodontitis with a high risk for tooth loss (Eke et al., 2020). The objective of this study was to test the hypothesis that the poorer periodontal status at the beginning of treatment of MDD with SSRI, is associated with the less favorable treatment outcomes independently of different socio‐demographic and clinical parameters.

## 2 METHODS

### 2.1 Study design

We performed this prospective cohort study from July 2018 to January 2019 at Psychiatric Hospital “Sveti Ivan”, Zagreb, Croatia. The study protocol was approved by the Ethics Committees of the School of Dental Medicine, University of Zagreb, and Psychiatric Hospital “Sveti Ivan.” We obtained informed consent from all patients and protected their anonymity by keeping the informed‐consent forms separate from the data collection instruments. We performed the study in accordance with the World Medical Association Declaration of Helsinki of 1975, as revised in 2013 (World Medical Association, 2013). Data are available at Mendeley public repository DOI: 10.17632/4sxh7dcss4.2.

### 2.2 Study population

The targeted population was patients diagnosed with MDD (ICD‐10 F32, F33) (The World Health Organization, 1990) who were treated in a psychiatric hospital with SSRI. Inclusion criteria were both genders, age 30 to 75 years, first treatment in a psychiatric hospital by SSRI during the current MDD episode. We chose the targeted age range of 30 to 75 years because the risk of having periodontal disease under the age of 30 is low (Araújo et al., 2016; Dumitrescu, 2016), and above the age of 75 years the number of somatic comorbidities that may confound our conclusions is probably too high. Exclusion criteria were: acute suicidality; schizophrenia spectrum disorders; and addiction (not counting smoking); and neurological, endocrinological, or other somatic conditions that may have an inflammatory component and therefore confound the results of the study (Karakelides et al., 2010; Loftis et al., 2008, 2010; Stewart et al., 2009).

### 2.3 The needed sample size and the sample type

We determined the targeted level of statistical significance at *p* < 0.05 and the targeted statistical power at 80%. We calculated the needed sample size before the data collection in order to detect the independent contribution of CAL to the prediction of HAM‐D17 at third month defined as the minimum increase in multiple coefficients of determination of *R* ^*2*^ = 0.15 (standardized effect size, *f* ^*2*^ ≈ 0.18), after the adjustment for eight covariates by linear multivariable regression. Under these conditions, a sample size of 38 was required. Expecting up to 10% of patients lost for follow‐up, we determined the initially needed sample size at 43. We performed the calculation using the PASS 14 Power Analysis and Sample Size Software (NCSS, LLC. Kaysville, UT, ncss.com/software/pass., 2015). We selected a consecutive sample of patients in order of their admission to the hospital.

### 2.4 Outcome

The outcome was change in the hamilton depression rating scale (HAM‐D17) (Hamilton, 1960) score after 3 months of treatment with SSRI, assessed by an experienced and trained psychiatrist (S.J.) during the semi‐structured interviews conducted at enrollment and at the control exam at 3‐month follow‐up. The HAM‐D17 score was computed as the sum of 17 items, each measuring the severity of particular MDD symptoms on the five‐ (eight items) or three‐ (nine items) point scale. Five‐point items range from “absent” to “severe,” and the three‐point items from “absent” to “clearly present.” During the psychiatric assessment, the psychiatrist was blinded to the patient's periodontal status. We did not independently reassess the HAM‐D17 results.

### 2.5 Independent variable (predictor)

The independent variable was CAL at baseline. We computed the CAL by adding together the periodontal pocket depths and gingival recession. We measured the pocket depths as the distance from the edge of the gingiva to the bottom of the sulcus or the periodontal pocket, in millimeters. We defined the gingival recession as the distance from the gingival cementoenamel junction to the free edge of the gingiva, in millimeters. We performed the measurement of periodontal pocket depths and gingival recession using Williams PCP 12 (PCP 10‐SE, Hu‐Friedy Mfg. Co. Inc., Chicago, IL) on four spots on each tooth. We rounded the depth of the probes to the nearest whole millimeter. The periodontal exam was performed by experienced periodontists (A.A.) and his colleagues from the Department of Periodontology, School of Dental Medicine, University of Zagreb, Croatia, who were all blinded to the patients' general psychiatric statuses, diagnoses, and severity of MDD. We did the periodontal exams once and did not repeat them independently. Each patient was examined only once, by only one periodontist, and we could not present the inter−/intra‐examiner reliability.

### 2.6 Possible confounders

After the analysis of the minimal sufficient adjustment for confounding using the direct acyclic graph as implemented in DAGitty v. 3.0 (Textor et al., 2016), and to avoid multicollinearity, we excluded the age at MDD onset because we already planned the adjustment for age and the duration of MDD. Finally, by multivariable analysis we controlled the possible confounding effects of baseline value of HAM‐D17, age, gender, body mass index (kg/m^2^), regular current smoking of tobacco, education, work status, having a steady life‐partner, monthly income per household member, diagnosis (ICD‐10: F32 or F33), duration of MDD, duration of current MDD episode, having a chronic physical illness, antidepressants daily dosage in fluoxetine‐equivalents and treatment with benzodiazepines. We calculated the fluoxetine‐equivalent daily doses by multiplying the doses of escitalopram by 2.22, sertraline by 0.41, paroxetine by 1.18, fluvoxamine by 0.28, maprotiline by 0.34, mirtazapine by 0.79, and venlafaxine by 0.27 (Hayasaka et al., 2015). We obtained the data on diagnosis and duration of MDD from the hospital medical records, and the data on age, education, work status, and monthly income per household member using the patients' self‐administered paper questionnaire at the enrollment, immediately after the psychiatric interview. For the description of our sample characteristics, we additionally collected data on number of previous MDD episodes, general functioning using the global assessment of functioning scale (Pedersen et al., 2018), treatment with specific antidepressants, and treatment with psychotherapy.

### 2.7 Statistical analysis

In the introductory, bivariable analysis of the change in depression severity during the three‐month treatment with SSRI, we calculated the means of absolute differences between the baseline and measurement after 3 months. Then we calculated the, means of relative differences as the absolute difference divided by the baseline value, and finally the standardized effect size: Morris & DeShon *d*_*RM*_ on the pooled standard deviation (Morris & DeShon, 2002). We calculated the statistical significance of the differences using the *t* test for correlated samples.

We tested the hypothesis using multivariable, hierarchical quadratic regression. We assessed the multivariate normality by inspecting the residuals histogram and *P–P* plot using the Shapiro–Wilk test on residuals, and homoscedasticity by analyzing the scatter diagram of regression standardized residuals and predicted values of HAM‐D17 scale after 3 months of treatment with SSRI. In the first hierarchical regression step we entered the baseline HAM‐D17 score. In the second step we included 14 covariates whose confounding effects we wished to control for. In the third step we included the linear CAL term centered by subtracting the sample means from each score, and the squared CAL sample‐mean‐centered as well. For each step we presented the unadjusted (*R* ^*2*^) and coefficient of determination adjusted for the number of predictors (*R* ^*2*^_*adj*_), change of the adjusted *R* ^*2*^ from the previous hierarchical step, F ratio of additionally explained and unexplained variance with its degrees of freedom and the statistical significance of its difference from zero. All statistical tests were two‐tailed with statistical significance set at *p* < 0.05. We corrected the *p*‐values for multiple testing using the Benjamini‐Hochberg method with false discovery rate (*FDR*) set at <5%. We performed the statistical data analysis using StataCorp 2019 (Stata Statistical Software: Release 16. College Station, TX: StataCorp LLC).

## 3 RESULTS

**Figure 1:**
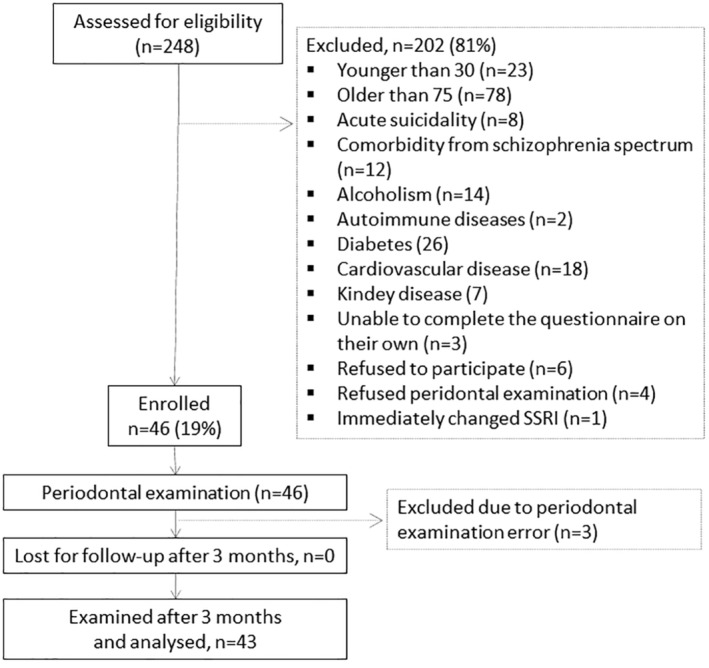
Participants flow; only the first reasons for exclusion are presented

We assessed 248 patients for eligibility (Figure 1). After the exclusion of 202 for not meeting the eligibility criteria and three patients due to periodontal examination errors, we examined and analyzed 43 patients diagnosed with MDD (ICD‐10 F32, F33) with a median (*IQR*) age of 50 (41–56) years, 34 (79%) of them women (Table 1). At baseline, at the introduction of therapy with SSRI, median (IQR) number of teeth was 23 (18–28), mean (*SD*) periodontal pocket depth was 3.4 (0.52) mm, ranging from 2.6 to 4.7 mm; and the mean (*SD*) gingival recession was 0.9 (0.59), ranging from 0 to 2.1 mm. Two periodontal indices were not significantly correlated (*r* = −0.08; CI_*95%*_ −0.37–0.16; *p* = 0.604; *FDR > 5%*). Baseline CAL was mean (*SD*) 4.3 (0.75) mm, ranging from 2.9 to 6.0 mm. All patients had some sings of periodontitis, 31 (72%) moderate, and 12 (27%) severe form. No patient was lost for follow‐up. After 3 months of therapy with SSRI, mean HAM‐D17 score was significantly lowered for three points (CI_*95%*_ −5.0 to −1.7) from mean (*SD*) of 19 (4.7) to 16 (5.4), what was the mean improvement of 15%, standardized effect size *d*_*RM*_ = −0.56 (*CI*_*95%*_ −1.03 to −0.17; *p* < 0.001; *FDR <* 5%).

**Table 1:** Patients' baseline characteristics (*n* = 43)

	*n* (%)
**Sociodemographic and vital characteristics**	
Female gender	34 (79)
Age (years), *median* (*IQR*)	50 (41–56)
Education	
Primary or secondary school	31 (72)
University	12 (28)
Having a steady life‐partner	25 (58)
Number of household members, *median* (*IQR*)	3 (2–4)
Work status	
Unemployed, retired or on the long sick‐leave	22 (51)
Employed	21 (49)
Monthly income per household member (EUR), *median* (*IQR*)	312 (177–401)
Body mass index (kg/m^2^), *median* (*IQR*)	25 (23–28)
Current smoking of tobacco	26 (61)
**Clinical characteristics**	
Diagnosis	
Depressive episode (ICD‐10F32)	13 (30)
Recurrent depressive disorder (ICD‐10F33)	30 (70)
Age at the first onset of depression (years), *median* (*IQR*)	44 (33–49)
Duration of MDD (years), *median* (*IQR*)	5 (1–11)
Number of previous MDD episodes, *median* (*IQR*)	2 (1–4)
Duration of the current episode (months), *median* (*IQR*)	3 (2–4)
Global assessment of functioning, *median* (*IQR*)	58 (54–64)
Having a chronic physical illness	19 (44)
Therapy	
Escitalopram	15 (35)
Sertraline	12 (28)
Paroxetine	9 (21)
Fluoxetine	5 (12)
Other^a^	5 (12)
Daily dosage (fluoxetine equivalent), *median* (*IQR*)	22 (20–35)
Other therapy	
Benzodiazepines	34 (79)
Psychotherapy	19 (44)
HAM‐D17 at baseline, *mean* (SD)	19 (4.7)
CAL (mm) at baseline, *mean* (SD)	4.3 (0.75)

*Note*: Data are presented as number (percentage) of participants if not stated otherwise.

Abbreviations: CAL, clinical attachment loss; HAM‐D17, Hamilton Depression Rating Scale‐17; *IQR*, interquartile range.

^a^ Other antidepressants, prescribed to one patient each were: fluvoxamine, maprotiline, tianeptine, mirtazapine, venlafaxine.

**Figure 2:**
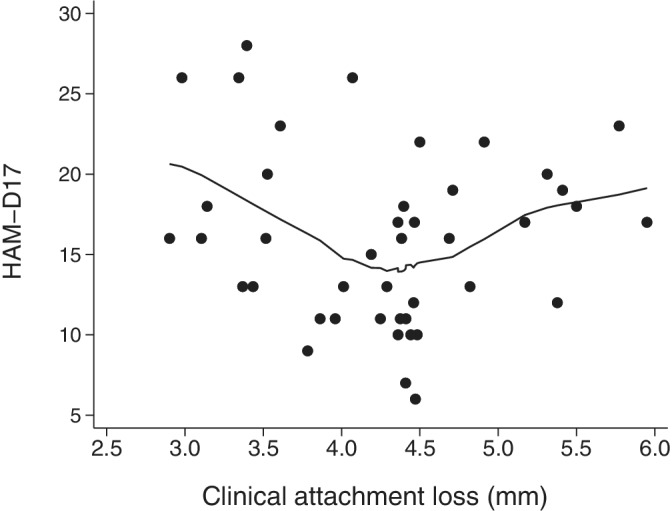
Scatter diagram of the correlation between baseline clinical attachment loss and the severity of depression after 3 months of treatment with SSRI, measured by Hamilton depression rating Scale‐17; line represent LOESS smoothing line with 80% span (*n* = 43)

Baseline HAM‐D17 result was a significant predictor of HAM‐D17 score 3 months later (*R* ^*2*^ = 0.19; *R* ^*2*^_*adj*_ = 0.17; *F*[1,41] = 9.70; *p* = 0.003; *FDR >* 5%) (Table 2). In the introductory analysis adjusted only for the baseline HAM‐D17 sore, the linear CAL term was not significant predictor of the HAM‐D17 after 3 months of treatment with SSRI (b = −1.07; CI_*95%*_ −3.01; 0.87; *p* = 0.273; FDR > 5%), but the CAL quadratic term was (b = 2.6; CI_*95%*_ 0.48; 4.76; *p* = 0.018; FDR < 5%). Introduction of 14 preplanned sociodemographic and clinical confounders did not significantly improve the prediction of HAM‐D17 at the third month of follow‐up (*R* ^*2*^ = 0.46; *R* ^*2*^_*adj*_ = 0.15; (*F*[14,27] = 0.9; *p* = 0.534; *FDR >* 5%). The introduction of the linear CAL term at the third step did not significantly improve the prediction of HAM‐D17 result (*R* ^*2*^ = 0.50; *R* ^*2*^_*adj*_ = 0.19; adjusted *R* ^*2*^ change = 0.04; *F*[1,26] = 2.1; *p* = 0.157; *FDR >* 5%). Finally, the introduction of the quadratic CAL term at the fourth step significantly improved the prediction of HAM‐D17 result (*R* ^*2*^ = 0.59; *R* ^*2*^_*adj*_ = 0.31; adjusted *R* ^*2*^ change = 0.12; *F*[1,25] = 5.5; *p* = 0.027; *FDR < 5%*) (Table 2). The quadratic curve turning point (vertex) was detected at the value of CAL = 4.44 mm (CI_*95%*_ 4.12 to 4.76 mm) (Figure 2). In patients with the baseline CAL bellow this value, the severity of MDD symptoms was significantly improved. In patients with the baseline CAL ≥4.44 mm, indicating the worse periodontal status, further increase in baseline CAL was associated with the significant worsening of MDD treatment outcomes independently of the baseline depression severity and 14 sociodemographic and clinical predictors of treatment outcome. In our sample there were 17 (40%; CI_*95%*_ 26%; 55%) of patients with CAL≥4.44 mm. Since a higher CAL value can occur due to the gingival recession and not only due to the periodontal pocket formation we additionally analyzed the value of baseline pocket depths and gingival recession in the prediction of HAM‐D17 score at the third month of follow‐up. After the adjustment for baseline HAM‐D17 score and all planned covariates, pocket depths were not statistically significantly associated with a reduction in the severity of depressive symptoms (*R* ^*2*^= 0.46; *R* ^*2*^_*adj*_ = 0.12; adjusted *R* ^*2*^ change = −0.03; *F*[1,26] = 0.14; *p* = 0.711; *FDR >* 5%), but the gingival recession was (*R* ^*2*^ = 0.55; *R* ^*2*^_*adj*_= 0.27; adjusted *R* ^*2*^ change = 0.11; *F*[1,26] = 5.17; *p* = 0.031; *FDR <* 5%).

**Table 2:** Multivariable hierarchical regression of Hamilton Depression Rating Scale result after 3 months of treatment with SSRI (*n* = 43)

	R^2^	R^2^_adj_	R^2^_adj change_	F(df)_change_	*p*
1st step					
HAM‐D17 at baseline	0.19	0.17	0.17	9.7 (1, 41)	0.003^b^
2nd step					
HAM‐D17 at baseline + 14 covariates^a^	0.46	0.15	−0.02	0.9 (14, 27)	0.534
3rd step					
HAM‐D17 at baseline + 14 covariates^a^ + CAL linear term	0.50	0.19	0.04	2.1 (1, 26)	0.157
4th step					
HAM‐D17 at baseline + 14 covariates^a^+ CAL linear term + CAL quadratic term	0.59	0.31	0.12	5.5 (1, 25)	0.027^b^

Abbreviation: HAM‐D17, Hamilton Depression Rating Scale‐17; CAL, Clinical attachment loss; *R*, coefficient of multiple correlation; *R* ^*2*^, coefficient of multiple determination; *R* ^*2*^_*adj*_, coefficient of multiple determination adjusted for number of predictors; *R* ^*2*^_*adj change*_, change of R^2^_adj_ from the previous step; *F(df)*, F ratio of this step additionally explained and unexplained variance with its degrees of freedom; *p*, statistical significance of the R^2^ change.

^a^ Covariates entered in this step were age, gender, body mass index, regular current smoking of tobacco, education, work status, having a steady life‐partner; monthly income per household member, diagnosis (ICD‐10: F32 or F33), duration of MDD, duration of current MDD episode, having a chronic physical illness, antidepressants daily dosage in fluoxetine‐equivalents and treatment with benzodiazepines.

^b^ FDR <5%.

## 4 DISCUSSION

We found significant and clinically relevant nonlinear association of baseline periodontal status with the outcome of MDD treatment with SSRI independently of 14 different sociodemographic and clinical parameters. After the CAL value of 4.44 mm, further worsening of periodontal status was associated with significant and clinically relevant worsening of MDD treatment outcomes.

Periodontitis and depression are multifactorial and long‐lasting diseases. They affect hypothalamic–pituitary–adrenal (HHA) axis dysregulation, cause an increase in cortisol levels, and by the effect on the neuroendocrine activity cause an increase in proinflammatory cytokines (Warren et al., 2014). Regulation of HHA axis is a prerequisite for recovery and a favorable therapeutic outcome of antidepressants (Belzung & Billette de Villemeur, 2010). Antidepressants reduce the plasma concentration of proinflammatory cytokines (Loftis et al., 2010), inflammatory processes moderated by cytokine concentrations in the brain (Pasquini et al., 2014), and lead to the normalization of HHA axis activities (Halaris, 2019). Proinflammatory cytokines IL‐6 and TNF‐α play a key role in the activation of the HHA axis (Elenkov, 2008), affecting tryptophan metabolism and decreased serotonin synthesis (Martinac et al., 2017). Conditions associated with elevated levels of proinflammatory cytokines, such as periodontitis, can alter the effect of SSRI indirectly by the decrease of serotonin activity in the brain (Hannestad et al., 2011). Periodontal pathogen endotoxins are associated with elevated inflammatory parameters and proinflammatory cytokines (especially TNF α and IL‐6) that may potentiate the inflammation and increase vulnerability to depression (Dumitrescu, 2016). Furthermore, a recent pre‐clinical in vivo study found the possible direct invasion of *Fusobacterium nucleatum*, but not *Porphyromonas gingivalis*, into the brain of Wistar:Han rats to whom periodontitis and chronic mild stress were induced, and thus indicating the possibility of neuroinflammation directly caused by the periodontal pathogens translocation through the blood–brain barrier (Martínez et al., 2021). It seems that periodontal disease is primarily associated with depression through the inflammatory mechanism and by the effects of cortisol concentrations to the immune system, rather than by psychosocial effects or quality of life (Cakmak et al., 2016). If the bidirectional hypothesis is true (Dumitrescu, 2016), the worse the periodontal condition is, the stronger the effect on MDD treatment outcomes that can be expected. This was indeed what we observed. Antibodies in serum and in cervical fluid are elevated only at the stage of advanced periodontal disease, preventing and delaying the disease progression, but once the protective mechanisms are overcome, a more pronounced destructive process and severity of periodontal disease begin (Haffajee et al., 1995). Studies examining serum cortisol levels in various forms of periodontitis have found that cortisol levels are significantly increased in the patients with aggressive periodontitis compared to those with chronic periodontitis (Haririan et al., 2012). The effect of antidepressants is better in patients with adrenocorticotropic hormone (ACTH) concentration below the population median ACTH (Brouwer et al., 2006). Thus, chronic periodontitis may also contribute to the severity of depression, and if this dysregulation is larger, as in chronic or aggressive periodontitis, it may possibly reduce the effectiveness of SSRI. Future studies on larger samples should include other MDD treatments, use the more specific treatment outcomes, primarily different dimensions of MDD, and check the hypothesis on the mediating effect of inflammation and elevated cytokines on the association between periodontal disease and the MDD treatment outcomes.

### 4.1 Limitations of the study

The primary limitation of our study was the lower sample size which forced us to use the HAM‐D total score as the outcome although the scale is not unidimensional and understanding of MDD as the consistent and unique syndrome is questionable. Our study was not powered for the assessment of the HAM‐D17 dimensions, and this may jeopardize the internal validity of our findings. The second limitation was that we selected a consecutive instead of random sample of patients, which might have increased the risk of sampling bias. For this reason, our sample may be biased toward the subpopulation of patients with more severe MDD symptoms, better access to psychiatric healthcare, or better insight, as these patients present more frequently in the psychiatric hospital. The third limitation was that we measured CAL only at baseline, at the introduction of therapy with SSRI, so we were not able to include the eventual later changes of periodontal status nor the possible periodontal treatment effects, while the patients suspected to have periodontal disease were advised to take a comprehensive periodontal exam, which possibly led to the periodontal treatment, and what would not have happened in a real‐life clinical setting, where periodontal disease often remains undetected. The probable effect of this limitation was in favor of our null hypothesis of no CAL effects on the poorer MDD treatment outcomes. Therefore, this limitation might not jeopardize the internal validity and direction of our conclusions but only lower the generalizability of our findings. The fourth limitation was that the reliability of periodontal pocket depths and gingival recession measurements are not perfect, and in our study they were estimated by different dental medicine physicians with no repeated measurements. As each patient was examined by only one person, we could not evaluate the reliability and validity of their assessments, nor could we determine the most probable direction and the extent of possibly so‐caused bias. Fifth, it was documented that HAM‐D17 often does not satisfy the temporal measurement invariance as the basic assumption for the validity of comparison of its score at baseline and at third month. It is possible that the observed change in total HAM‐D17 score after 3 months of treatment represents the change in the structure of symptoms and not the lowering of their overall severity, as it is possible that the baseline CAL is associated with specific MDD symptom dimensions and not with its overall ease. Sixth, we performed the study in a large psychiatric hospital in a large city, and our findings should only cautiously be generalized to the population of MDD patients treated in smaller institutions, general hospitals, private practices, and in more rural areas.

### 4.2 Conclusion

It seems that the outcome of MDD treatment with SSRI is associated with the patients' baseline periodontal status. Periodontal healthcare is accessible, and we may utilize it in an integrative, multidisciplinary approach not only for the sake of patients' quality of life and prevention of periodontal disease, but for the sake of the outcomes of psychiatric treatment as well.

## 5 CLINICAL RELEVANCE

### 5.1 Scientific rationale for the study

Periodontal disease as the systemic inflammation condition may play an important role in the pathogenesis of major depressive disorder but the possible effect of the periodontal status to the depression treatment outcomes is unknown.

### 5.2 Principal findings

The poorer baseline periodontal status is associated with the less favorable outcome of treatment of depression, independently of various sociodemographic, and clinical confounding factors.

### 5.3 Practical implications

Prevention and treatment of periodontal disease may improve the major depressive disorder treatment outcomes and should be utilized as the standard of mental healthcare.

## CONFLICT OF INTEREST

The authors declare no potential conflict of interest.

## AUTHOR CONTRIBUTION

Conception and design: Silvana Jelavić, Žarko Bajić, Ivona Šimunović Filipčić, Ivana Jurčić Čulina, Igor Filipčić, Andrej Aurer; Data collection: Silvana Jelavić, Andrej Aurer; Data analysis and interpretation: Silvana Jelavić, Žarko Bajić, Ivona Šimunović Filipčić, Drafting the manuscript: Silvana Jelavić, Žarko Bajić, Ivana Jurčić Čulina, Revising the manuscript critically: Ivona Šimunović Filipčić, Ivana Jurčić Čulina, Igor Filipčić, Andrej Aurer, Final reading and approve of the manuscript: All authors.

## Data Availability

Availability of data and material Data are available at Mendeley public repository, DOI: 10.17632/4sxh7dcss4.2. Code availability STATA code is available from the corresponding author upon request.
